# Association between estimated glomerular filtration rate and reversion to normoglycemia in people with impaired fasting glucose: a 5-year retrospective cohort study

**DOI:** 10.1186/s40001-024-01669-y

**Published:** 2024-02-22

**Authors:** Lirong Tu, Haofei Hu, Xinglei Zhou, Heping Zhang, Xiaohui Liu, Dehua Yang, Yongcheng He

**Affiliations:** 1 Department of Nephrology, Affiliated Hospital of North Sichuan Medical College, No.1 Maoyuan South Rd, Nanchong, 637000 Sichuan Province China; 2 Department of Nephrology, The First Affiliated Hospital of Shenzhen University, Shenzhen, 518000 Guangdong Province China; 3 Department of Nephrology, Shenzhen Second People’s Hospital, Shenzhen, 518000 Guangdong Province China; 4 Department of Nephrology, Second Affiliated Hospital of Xuzhou Medical University, Xuzhou, 221006 Jiangsu Province China; 5 Department of Pediatrics, Shenzhen Hengsheng Hospital, No. 20 Yintian Road, Baoan District, Shenzhen, 518103 Guangdong Province China; 6 Department of Nephrology, Shenzhen Hengsheng Hospital, Shenzhen, 518103 Guangdong Province China

**Keywords:** Impaired fasting glucose, Regression to normoglycemia, Cox proportional-hazards regression model, Competitive risk model, Nonlinear relationship

## Abstract

**Objectives:**

The present body of evidence regarding the correlation between the estimated glomerular filtration rate (eGFR) and the reversal of impaired fasting glucose (IFG) to normoglycemia remains constrained. Consequently, the objective of our study is to examine the relationship between eGFR and the restoration of normoglycemia in individuals with IFG.

**Methods:**

This retrospective cohort study consecutively collected data from 24,541 non-selective participants with IFG at Rich Healthcare Group in China from January 2010 to 2016. We aimed to investigate the association between baseline eGFR and reversion to normoglycemia using the Cox proportional-hazards regression model. Through the utilization of a Cox proportional hazards regression model featuring cubical spline smoothing, we were able to ascertain the non-linear correlation between eGFR and the return to normoglycemia. Furthermore, various sensitivity and subgroup analyses were carried out, and a competing risk multivariate Cox regression was employed to examine the progression to diabetes as a competing risk for the reversal of normoglycemic events.

**Results:**

In our study, comprising 24,541 participants, the average age was 49.25 ± 13.77 years, with 66.28% being male. The baseline eGFR mean was 104.16 ± 15.78 ml/min per 1.73 m^2^. During a median follow-up period of 2.89 years, we observed a reversion rate to normoglycemia of 45.50%. Upon controlling for covariates, our findings indicated a positive correlation between eGFR and the probability of returning to normoglycemia (HR = 1.008, 95% CI 1.006–1.009). In addition, a non-linear association was observed between eGFR and the likelihood of transitioning from IFG to normoglycemia. The inflection point of eGFR was found to be 111.962 ml/min per 1.73 m^2^, with HRs of 1.003 (95% CI 1.001, 1.005) on the left side of the point and 1.019 (95% CI 1.015, 1.022) on the right side. Our robust results were supported by competing risks multivariate Cox's regression and sensitivity analysis.

**Conclusions:**

The findings of our investigation indicate a favorable and non-linear correlation between eGFR and the restoration of normoglycemia in Chinese individuals with IFG. Specifically, a reduction in renal function at an early stage in these patients may considerably diminish the likelihood of attaining normoglycemia.

**Supplementary Information:**

The online version contains supplementary material available at 10.1186/s40001-024-01669-y.

## Introduction

Diabetes is a significant public health concern due to its high prevalence, mortality rates, and escalating costs [1]. Pre-diabetes is characterized by a state of hyperglycemia, where blood glucose levels are elevated but not as high as those observed in individuals with diabetes [2]. According to the International Diabetes Federation (IDF) in 2017, approximately 374 million adults worldwide were diagnosed with pre-diabetes, and this figure is projected to increase to 548 million by 2045, accounting for 8.4% of the adult population [3]. The estimated prevalence of pre-diabetes among Chinese adults is approximately 35.7% [4]. Although 5–10% of individuals with pre-diabetes progress to diabetes mellitus (DM) annually [5], a significant proportion of them, ranging from 20% to 50%, may revert to normoglycemia [6–8]. It is noteworthy that pre-diabetes elevates the risk of not only developing type 2 diabetes mellitus (T2DM), but also of experiencing cardiovascular disease and microvascular complications [9–11]. Previous research has demonstrated that a brief return to normoglycemia is linked to a notable reduction in the likelihood of developing type 2 diabetes in individuals with pre-diabetes, highlighting the advantageous clinical implications of this outcome [12]. As such, the principal objective of screening and treating pre-diabetes should be to restore patients to a normoglycemic state.

While disease progression is a significant concern in clinical settings, identifying contributing factors for pre-diabetes regression to normoglycemia is equally, if not more important, as it can help prevent pre-diabetes from developing into diabetes and inform actionable targets for maintaining public health efforts. However, few studies have examined reversion rates and associated contributing factors for individuals with pre-diabetes. Previous epidemiological studies have provided evidence indicating that regression to normoglycemia may be related to various factors, including age, insulin secretion, beta-cell function, baseline fasting plasma glucose (FPG), obesity, and fasting triglycerides [13–16].

The diagnosis of chronic kidney disease (CKD) is commonly established through the utilization of the estimated glomerular filtration rate (eGFR), which serves as a precise indicator of renal filtration function [17]. A recent meta-analysis encompassing eight cohort studies employed impaired fasting plasma glucose (FPG) as a criterion for pre-diabetes, revealed a slightly elevated likelihood of impaired renal function linked to impaired FPG [18]. In addition, a Chinese study found that, in healthy individuals, FPG (but not 2-h post-load blood glucose or glycated hemoglobin A1c) was linked to a mild decline in eGFR [19]. Prior community-based investigations in China have demonstrated an inverse and non-linear correlation between eGFR and the hazards of diabetes and pre-diabetes [20, 21]. Nevertheless, extant literature does not provide any indication of a relationship between eGFR (both lower and higher levels) and pre-diabetes regression to normoglycemia. Since impaired fasting glucose (IFG) is the main representative of pre-diabetes, we conducted a cohort study to explore the association between eGFR and the probability of IFG reversion to normoglycemia in the Chinese community.

## Methods

### Study design

A retrospective cohort study was conducted utilizing data from the China Rich Healthcare Group database to investigate the correlation between eGFR and IFG reversion to normoglycemia. The analysis centered on eGFR at baseline as the independent variable of interest, while reversion to normoglycemia at follow-up was considered the dependent variable.

### Data source

The raw data utilized in this study was obtained from the DATADRYAD database (https://datadryad.org/stash) at no cost, as made available by Chen, Ying et al. [22]. Data from: association of body mass index and age with incident diabetes in Chinese adults: a population-based cohort study, Dryad, data set, https://doi.org/10.5061/dryad.ft8750v. According to the terms of service of the Dryad database, researchers are permitted to use the data set for non-commercial purposes and may share, remix, modify and create derivative works from it, provided that proper credit is given to the author and source [22].

### Study population

Selection bias was identified as the most frequent type of bias in our study, which could have resulted in over- or underestimation of the results. To minimize the impact of selection bias, the Rich Healthcare Group conducted non-selective and consecutive collection of participant data from 32 locations in 11 cities across China. To safeguard the confidentiality of the participants, their identifying information was encoded as non-traceable codes. The data utilized in this study were sourced from a computerized database established by the Rich Healthcare Group in China, which comprehensively documented the medical records of individuals who underwent health assessments between 2010 and 2016. The study was initially sanctioned by the Rich Healthcare Group Review Board, and due to the retrospective nature of the study, informed consent was deemed unnecessary and thus waived [22, 23].

**Fig. 1:**
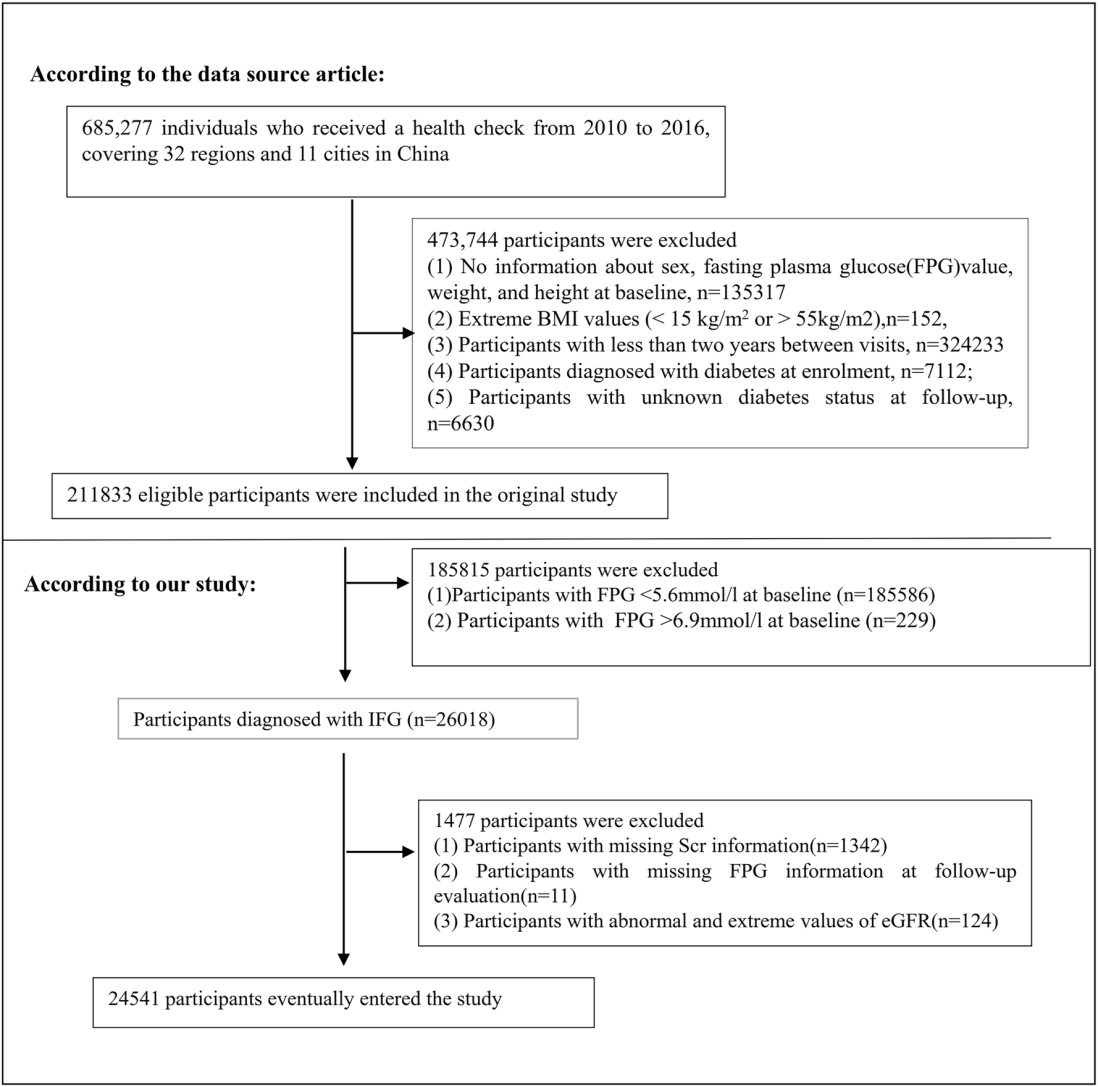
Flowchart of study participants. The selection process of participants is depicted in this figure. The original study assessed 211,833 individuals for eligibility, from which 187,292 were excluded. The present study's final analysis comprised 24,541 subjects

The original study initially enrolled a total of 685,277 participants aged 20 or above who had undergone at least two health exams. Of these, 473,444 participants were excluded based on the following criteria: (i) visit period of less than 2 years; (ii) extreme body mass index (BMI) values (15 kg/m^2^ or > 55 kg/m^2^); (iii) incomplete records of weight, sex, height, and FPG value at baseline; (iv) diagnosis of diabetes at enrollment; and (v) unknown diabetes status at follow-up. After applying these exclusion criteria, the analysis was conducted on a total of 211,833 people [22]. In the present investigation, we proceeded to eliminate an additional 185,815 subjects who exhibited baseline FPG levels that fell below 5.6 mmol/L or exceeded 6.9 mmol/L. Furthermore, we excluded participants who lacked serum creatinine (Scr) data at baseline (*n* = 1342), those who lacked FPG information at follow-up (*n* = 11), and those who displayed anomalous and extreme eGFR values (i.e., values that were greater or less than three standard deviations from the mean) (*n* = 124) [24]. After these exclusions, 24,541 participants were included in the current study. The selection process is visually presented in Fig. 1. It is worth noting that according to the 2022 criteria of the American Diabetes Association, pre-diabetes is defined as the presence of impaired fasting glucose (IFG) (FPG level of 5.6–6.9 mmol/L), impaired glucose tolerance (IGT), and/or HbA1c (HbA1c level of 5.7–6.4%) [25]. In our study, we use IFG directly to represent pre-diabetes.

### Variables

#### Estimated glomerular filtration rate

We obtained information on eGFR at the beginning of the study and recorded it as a continuous variable. To calculate eGFR for "Asian origin" patients, we used the chronic kidney disease–epidemiology collaboration (CKD–EPI) equations, which take into account gender, age, and Scr [26]. The following formulas were used:

For females with Scr concentration ≤ 0.7 mg/dL,

$${\text{eGFR}} = {151} \times \left( {{\text{Scr}}/0.{7}} \right)^{{ - 0.{328}}} \times 0.{993}^{{{\text{age}}}} ;$$

For females with Scr concentration > 0.7 mg/dL,

$${\text{eGFR}} = {151} \times \left( {{\text{Scr}}/0.{7}} \right)^{{ - {1}.{21}0}} \times 0.{993}^{{{\text{age}}}} ;$$

For males with Scr concentration ≤ 0.9 mg/dL,

$${\text{eGFR}} = {149} \times \left( {{\text{Scr}}/0.{9}} \right)^{{ - 0.{415}}} \times 0.{993}^{{{\text{age}}}} ;$$

For males with Scr concentration > 0.9 mg/dL,

$${\text{eGFR}} = {149} \times \left( {{\text{Scr}}/0.{9}} \right)^{{ - {1}.{21}0}} \times 0.{993}^{{{\text{age}}}} ;$$

Age was measured in years, while Scr was measured in mg/dL. The utilization of the Asian-modified CKD–EPI equation facilitated a more precise assessment of GFR in Chinese populations exhibiting elevated GFR levels.

#### Outcome measures

Our intriguing outcome variable was the occurrence of reversion to normoglycemia, defined as an FPG < 5.6 mmol/l during follow-up assessment and no self-reported incidence of diabetes [25, 27].

#### Covariates

The study's covariates were selected based on the original study, previous research on factors influencing diabetes or pre-diabetes, and our clinical expertise [16, 20–22, 28]. The covariates included the following variables: (i) categorical variables: smoking status, sex, family history of diabetes, and drinking status. (ii) continuous variables: systolic blood pressure (SBP), BMI, aspartate aminotransferase (AST), high-density lipoprotein cholesterol (HDL-c), blood urea nitrogen (BUN), triglyceride (TG), diastolic blood pressure (DBP), FPG, total cholesterol (TC), alanine aminotransferase (ALT), and low-density lipid cholesterol (LDL-c).

#### Data collection

At every health check center visit, participants were provided with a detailed questionnaire regarding their lifestyle, family history of diabetes, personal medical history, and demographic characteristics. Trained staff measured height, blood pressure, and weight with precision and accuracy. To obtain accurate weight readings, participants wore light clothing and no shoes, and measurements were recorded to be within 0.1 kg. The BMI was computed through the division of weight (in kilograms) by the square of height (in meters). Height was precisely measured to within 0.1 cm. The blood pressure of participants was measured through office blood pressure measurements using standard mercury sphygmomanometers. Before the measurement, participants were instructed to rest quietly for 5–10 min while lying down. The smoking behavior of participants was classified into three categories, namely, currently smoking, ever smoking, and never smoking. Likewise, the drinking status of participants was categorized as currently drinking, ever drinking, or never drinking. The assessment of smoking and drinking status was conducted solely at baseline. At each visit, venous blood samples were collected following a minimum of 10 h of fasting. Subsequently, they were subjected to analysis for HDL-c, BUN, TC, AST, TG, Scr, FPG, LDL-c, and ALT utilizing an autoanalyzer (Beckman 5800) [22].

### Missing data processing

Of the participants in the study, a small percentage had missing data for certain variables. Specifically, 6 (0.0024%) participants were missing SBP and DBP data, 396 (1.61%) were missing TC and TG data. A much larger proportion of 9328 (38.01%), 8707 (35.48%), and 13,846 (56.42%) were missing HDL-c, LDL-c, and AST data, respectively, while 132 (0.54%) were missing ALT data and 1561 (6.36%) were missing BUN data. Moreover, 16,198 (66.00%) of the participants were missing data on both smoking and drinking status. To handle these missing data, multiple imputations were used for the covariates [29], with the imputation model, including BMI, HDL-c, age, gender, DBP, TC, SBP, LDL-c, ALT, BUN, TG, family history of diabetes, FPG, AST, and drinking and smoking status. We analyzed the missing data using missing-at-random (MAR) assumptions [30].

### Statistical analysis

The participants were stratified into quartiles of eGFR. Baseline characteristics for continuous variables were reported as mean ± standard deviation (SD) for Gaussian distribution or median (range) for skewed distribution, while categorical variables were reported as percentages. To assess differences among the various eGFR groups, three types of statistical tests were employed: one-way ANOVA for normal distribution, *χ*^2^ for categorical variables, or Kruskal–Wallis *H* for skewed distribution. The Kaplan–Meier method was utilized to determine survival estimates and time-to-event variables. Furthermore, the log-rank test was employed to assess the probability of reversion to normoglycemia from IFG across the eGFR groups.

To assess the possibility of covariate collinearity, the variance inflation factor (VIF) [31] was computed using the formula VIF = 1/(1 − *R*^2^), where *R*^2^ was the *R*-squared value derived from a linear regression equation. The variable under investigation was designated as the dependent variable, while all other variables were considered independent variables in each regression. Variables with a VIF greater than 5 were deemed collinear and were excluded from the multiple regression model (Additional file 1: Table S1).

Following collinearity screening, we employed univariate and multivariate Cox proportional hazards regression models to investigate the association between eGFR and the probability of reverting to normoglycemia from IFG. Model I was the unadjusted model with no controlled covariates. Model II was the model that underwent minimal adjustment, wherein only sociodemographic variables, namely, DBP, family history of diabetes, gender, SBP, smoking and drinking status, and BMI, were adjusted. On the other hand, Model III was the fully adjusted model, wherein covariates presented in Table 1, such as TG, gender, BUN, BMI, ALT, DBP, FPG, AST, HDL-c, SBP, family history of diabetes, LDL-c, smoking and drinking status, were taken into account. Effect sizes (HR) and 95% confidence intervals (CIs) were documented, while covariates were adjusted based on literature reports, clinical experience, and the outcomes of univariate analysis. The ultimate multivariate Cox proportional hazards equation omitted TC as a result of its collinearity with other variables, as presented in Additional file 1: Table S1.

**Table 1:** Baseline characteristics of participants

eGFR group	Q1 (< 94.07)	Q2 (94.07–105.10)	Q3 (105.10–115.75)	Q4 (≥ 115.75)	*P* value
Participants	6133	6137	6134	6137	
Age (years)	60.11 ± 13.37	54.36 ± 11.32	46.56 ± 8.92	35.96 ± 6.64	< 0.001
BMI (kg/m^2^)	25.17 ± 3.11	24.94 ± 3.21	24.84 ± 3.26	24.28 ± 3.78	< 0.001
SBP (mmHg)	132.22 ± 18.75	128.65 ± 17.56	125.50 ± 16.75	122.10 ± 15.40	< 0.001
DBP (mmHg)	79.61 ± 11.46	79.26 ± 10.94	78.56 ± 10.99	76.00 ± 10.78	< 0.001
FPG (mmol/L)	5.99 ± 0.33	5.97 ± 0.32	5.94 ± 0.32	5.89 ± 0.29	< 0.001
TC (mmol/L)	5.10 ± 0.95	5.11 ± 0.96	4.99 ± 0.93	4.72 ± 0.93	< 0.001
TG (mmol/L)	1.53 (1.07–2.24)	1.50 (1.03–2.19)	1.46 (0.99–2.20)	1.20 (0.81–1.88)	< 0.001
HDL-c (mmol/L)	1.33 ± 0.29	1.34 ± 0.32	1.32 ± 0.30	1.32 ± 0.30	< 0.001
LDL-c (mmol/L)	2.97 ± 0.71	2.98 ± 0.73	2.89 ± 0.71	2.72 ± 0.70	< 0.001
ALT (U/L)	21.30 (16.00–30.10)	22.00 (16.00–32.30)	23.20 (16.00–35.00)	22.00 (14.00–36.10)	< 0.001
AST (U/L)	25.00 (20.63–30.98)	24.88 (20.10–30.73)	24.50 (19.58–31.00)	23.11 (18.00–30.00)	< 0.001
BUN (mmol/L)	5.40 ± 1.24	5.09 ± 1.19	4.88 ± 1.17	4.55 ± 1.11	< 0.001
Scr (umol/L)	86.23 ± 13.41	73.19 ± 12.17	68.21 ± 11.81	62.33 ± 11.28	< 0.001
eGFR (mL/min·1.73 m^2^)	83.10 ± 8.64	99.85 ± 3.13	110.26 ± 3.07	123.40 ± 5.70	< 0.001
Gender					< 0.001
Male	4455 (72.64%)	4159 (67.77%)	4044 (65.93%)	3607 (58.77%)	
Female	1678 (27.36%)	1978 (32.23%)	2090 (34.07%)	2530 (41.23%)	
Smoking status					< 0.001
Never smoker	4237 (69.09%)	4293 (69.95%)	4466 (72.81%)	4852 (79.06%)	
Ever smoker	250 (4.08%)	275 (4.48%)	274 (4.47%)	275 (4.48%)	
Current smoker	1646 (26.84%)	1569 (25.57%)	1394 (22.73%)	1010 (16.46%)	
Drinking status					< 0.001
Never drinker	4948 (80.68%)	4955 (80.74%)	4909 (80.03%)	5140 (83.75%)	
Ever drinker	914 (14.90%)	951 (15.50%)	1001 (16.32%)	883 (14.39%)	
Current drinker	271 (4.42%)	231 (3.76%)	224 (3.65%)	114 (1.86%)	
Family history of diabetes					< 0.001
No	6035 (98.40%)	6011 (97.95%)	5963 (97.21%)	5914 (96.37%)	
Yes	98 (1.60%)	126 (2.05%)	171 (2.79%)	223 (3.63%)	

Values are *n* (%), mean ± SD or medians (quartiles)

*BMI* body mass index, *TC* total cholesterol, *DBP* diastolic blood pressure, *FPG* fasting plasma glucose, *TG* triglyceride, *ALT* alanine aminotransferase, *SBP* systolic blood pressure, *LDL-c* low-density lipid cholesterol, *AST* aspartate aminotransferase, *BUN* blood urea nitrogen, *eGFR* estimated glomerular filtration rate, *HDL-c* high-density lipoprotein cholesterol, *Scr* serum creatinine

Cox proportional hazards regression models have been criticized for being inadequate in handling non-linear models. In light of this, we have implemented the Cox proportional hazards model with cubic spline functions and smooth curve fitting to address non-linearity between eGFR and reversion to normoglycemia in individuals with IFG. Upon detecting non-linearity, a recursive algorithm was employed to identify the inflection point, followed by the implementation of two-piecewise Cox proportional hazards models on either side of the inflection point. The log-likelihood ratio test was utilized to determine the most suitable model [32].

Given that patients who develop diabetes during the follow-up period are less likely to revert to normoglycemia, this could potentially impede the detection of pre-diabetes reversal to normoglycemia or change the probability of such events [33, 34]. As a result, we performed a multivariate Cox proportional hazards regression analysis for competing risks, following the methodology outlined by Fine and Gray. In this analysis, the occurrence of diabetes was considered a competing risk for the occurrence of events leading to the reversal of normoglycemia [34, 35].

For subgroup analyses (gender, SBP, BMI, TG, age, drinking and smoking status, DBP, and family history of diabetes), we implemented a stratified Cox proportional hazards model. First, continuous variables, such as age (< 60, ≥ 60 years), SBP (< 140, ≥ 140 mmHg), TG (< 1.7, ≥ 1.7 mmol/L), BMI (< 18.5, ≥ 18.5 to < 24, ≥ 24 to 28, ≥ 28 kg/m^2^), and DBP (< 90, ≥ 90 mmHg), were transformed into categorical variables with clinical cutoff points [36–38]. Subsequently, all stratifications were controlled for other variables, excluding the stratification factor itself (TG, gender, BUN, BMI, FPG, ALT, DBP, AST, family history of diabetes, HDL-c, SBP, LDL-c, smoking and drinking status). Subsequently, a likelihood ratio test was conducted to scrutinize the existence of interactions in models that incorporated interaction terms and those that did not [39, 40].

To evaluate the robustness of our results, we conducted sensitivity analyses. Specifically, we categorized eGFR into quartiles and computed the *P* value for the trend to assess the reliability of eGFR as a continuous variable and explore potential non-linear relationships. A definition of decreased eGFR was established as a value below 90 ml/min/1.73 m^2^ [19]. The consumption of tobacco and alcohol has been linked to an elevated likelihood of developing T2DM [41]. In conducting additional sensitivity analyses to explore the correlation between eGFR and the return to normoglycemia in individuals with IFG, those with a history of smoking and drinking or an eGFR below 90 mL/min·1.73 m^2^ were excluded. As a sensitivity analysis, we excluded drinking and smoking status from the multivariate model due to their incomplete data in approximately 70% of cases and the potential lack of usefulness as covariates for model adjustment. In addition, we employed a generalized additive model (GAM) to incorporate the continuity covariate as a curve (model IV) to ensure result consistency [42]. Finally, *E* values were computed to evaluate the plausibility of unmeasured confounding between eGFR and reversion to normoglycemia [43]. The results were consistent with the guidelines outlined in the STROBE statement [44].

Empower Stats (X&Y Solutions, Inc., Boston, MA, http://www.empowerstats.com) and R statistical software packages (http://www.r-project.org, The R Foundation) were utilized for all analyses. Statistical significance was determined by two-sided *P* values below 0.05.

## Results

### Characteristics of participants

**Fig. 2:**
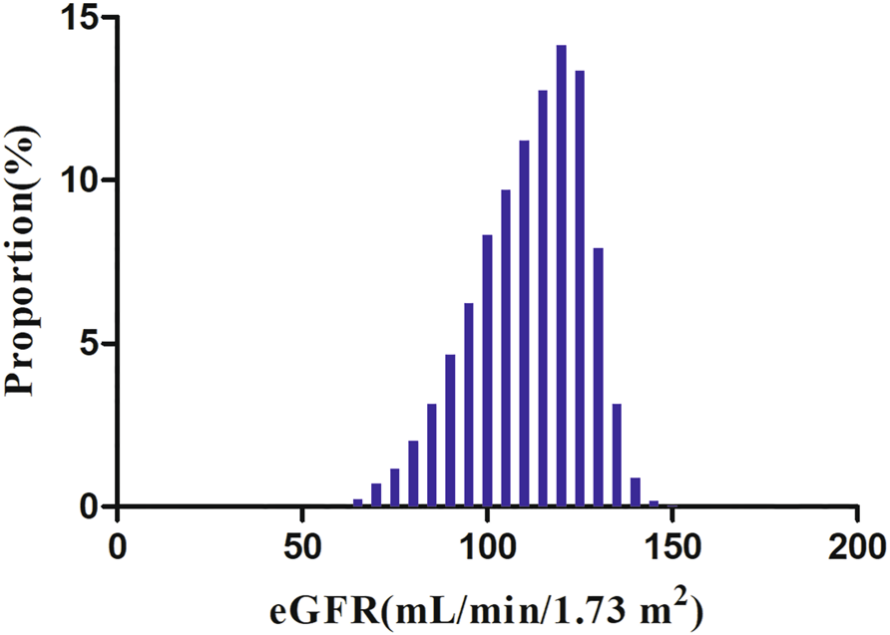
Distribution of eGFR. The normal distribution of eGFR depicted in this figure spans from 54.98 to 151.09 ml/min per 1.73 m^2^, with a mean value of 104.16 ml/min per 1.73 m^2^

Table 1 displays the demographic and clinical attributes of the individuals involved in the study. The individuals included in the study had a mean age of 49.25 ± 13.77 years, and 16,265 (66.28%) were male. Their baseline eGFR had a mean of 104.16 ± 15.78 ml/min per 1.73 m^2^. Among these individuals, 11,215 (45.50%) patients with IFG reverted to normoglycemia during a median follow-up of 2.89 years. The adults were grouped into subcategories based on eGFR quartiles (< 94.07, ≥ 94.07 to < 105.10, ≥ 105.10 to < 115.75, and ≥ 115.75). The Q4 group (eGFR ≥ 115.75 ml/min/1.73 m^2^) had significantly higher proportions of never-smokers, females, never-drinkers, and family history of diabetes compared to the Q1 (< 94.07 ml/min/1.73 m^2^) group. Conversely, the variables related to SBP, LDL-c, age, BMI, TG, FPG, TC, Scr, DBP, AST, current or ever smokers, BUN, ALT, males, and current or ever drinkers demonstrated opposite results. The normal distribution of eGFR levels, ranging from 54.98 to 151.09 ml/min per 1.73 m^2^ with an average of 104.16 ml/min per 1.73 m^2^, is depicted in Fig. 2.

### The reversal rate to normoglycemia from IFG

Of the participants with IFG, 11,215 individuals reverted to normoglycemia, resulting in a cumulative overall rate of 15.49 per 100 person-years. The cumulative reversal rate to normoglycemia ranged from 12.64 to 20.23 per 100 person-years across the four eGFR groups for participants with IFG. Specifically, the reversal rates for total normoglycemia and each eGFR group were 45.70% (45.08–46.32%), 37.42% (36.21–38.63%), 40.56% (39.33–41.79%), 46.15% (44.90–47.40%), and 58.66% (57.43–59.89%), respectively. Participants with higher eGFR showed higher rates of reversal to normoglycemia (*P* < 0.0001 for trend) (Table 2).

**Table 2:** Rate of reversion to normoglycemia in people with IFG

eGFR	Participants (*n*)	Reversion events (*n*)	Reversal rate (95% CI) (%)	Per 100 person-year
Total	24,541	11,215	45.70 (45.08–46.32)	15.49
Q1 (< 94.07)	6133	2295	37.42 (36.21–38.63)	12.64
Q2 (94.07–105.10)	6137	2489	40.56 (39.33–41.79)	13.66
Q3 (105.10–115.75)	6134	2831	46.15 (44.90–47.40)	15.51
Q4 (≥ 115.75)	6137	3600	58.66 (57.43–59.89)	20.23
*P* for trend			< 0.001	

eGFR, estimated glomerular filtration rate (mL/min·1.73 m^2^)

**Fig. 3:**
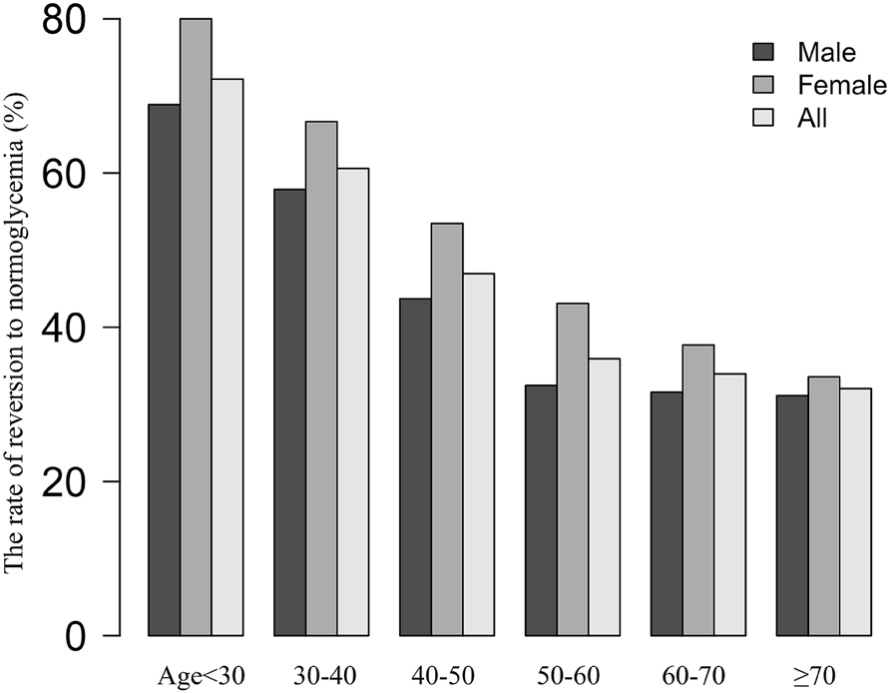
Rate of reversion to normoglycemia in patients with IFG of age stratification by 10 intervals. According to this figure, participants with IFG showed a higher rate of reversion to normoglycemia among women than men, regardless of their age group. Furthermore, the reversal rate in both men and women decreased with increasing age

In terms of age stratification by 10-year intervals, women had higher rates of reversion to normoglycemia than men across all age groups (Fig. 3). In addition, the reversal rate decreased with age for both men and women.

### Univariate Cox proportional hazards regression

Univariate analyses revealed that in patients with IFG, reversion to normoglycemia was negatively associated with age, TG, DBP, ALT, BMI, TC, SBP, LDL-c, AST, FPG, Scr, current or ever drinkers, BUN, current or ever smokers, and family history of diabetes. Conversely, it was positively related to HDL-c, females, never smokers, and never drinkers (all *P* < 0.05; see Table 3).

**Table 3:** Factors influencing reversion to normoglycemia among participants with IFG analyzed by univariate Cox proportional hazards regression

Variable	Statistics	HR (95%CI)	*P* value
Age (years)	49.248 ± 13.770	0.976 (0.974, 0.977)	< 0.00001
Gender			
Male	16,265 (66.277%)	Ref	
Female	8276 (33.723%)	1.267 (1.220, 1.317)	< 0.00001
BMI (kg/m^2^)	24.811 ± 3.367	0.934 (0.929, 0.940)	< 0.00001
SBP (mmHg)	127.116 ± 17.562	0.989 (0.988, 0.990)	< 0.00001
DBP (mmHg)	78.360 ± 11.134	0.985 (0.983, 0.986)	< 0.00001
FPG (mmol/L)	5.946 ± 0.318	0.222 (0.206, 0.240)	< 0.00001
TC (mmol/L)	4.982 ± 0.956	0.877 (0.860, 0.894)	< 0.00001
TG (mmol/L)	1.792 ± 1.444	0.891 (0.877, 0.905)	< 0.00001
HDL-c (mmol/L)	1.327 ± 0.304	1.669 (1.575, 1.769)	< 0.00001
LDL-c (mmol/L)	2.889 ± 0.720	0.911 (0.887, 0.935)	< 0.00001
ALT (U/L)	28.531 ± 23.621	0.993 (0.992, 0.994)	< 0.00001
AST (U/L)	26.365 ± 12.016	0.986 (0.985, 0.988)	< 0.00001
BUN (mmol/L)	4.980 ± 1.219	0.954 (0.939, 0.969)	< 0.00001
Scr (umol/L)	72.487 ± 15.046	0.997 (0.996, 0.998)	< 0.00001
eGFR (mL/min·1.73 m^2^)	104.157 ± 15.778	1.013 (1.011, 1.014)	< 0.00001
Smoking status			
Never smoker	17,848 (72.727%)	Ref.	
Ever smoker	1074 (4.376%)	0.882 (0.803, 0.968)	0.00838
Current smoker	5619 (22.896%)	0.783 (0.747, 0.820)	< 0.00001
Drinking status			
Never drinker	19,952 (81.301%)	Ref.	
Ever drinker	3749 (15.276%)	0.945 (0.897, 0.995)	0.03048
Current drinker	840 (3.423%)	0.779 (0.696, 0.871)	0.00001
Family history of diabetes			
No	23,923 (97.482%)	Ref.	
Yes	618 (2.518%)	0.760 (0.671, 0.861)	0.00002

*BMI* body mass index, *eGFR* estimated glomerular filtration rate, *FPG* fasting plasma glucose, *TG* triglyceride, *DBP* diastolic blood pressure, *AST* aspartate aminotransferase, *TC* total cholesterol, *ALT* alanine aminotransferase, *SBP* systolic blood pressure, *LDL-c* low-density lipid cholesterol, *Scr* serum creatinine, *HDL-c* high-density lipoprotein cholesterol, *BUN* blood urea nitrogen *HR* Hazard ratios, *Ref* reference, *CI* confidence interval

**Fig. 4:**
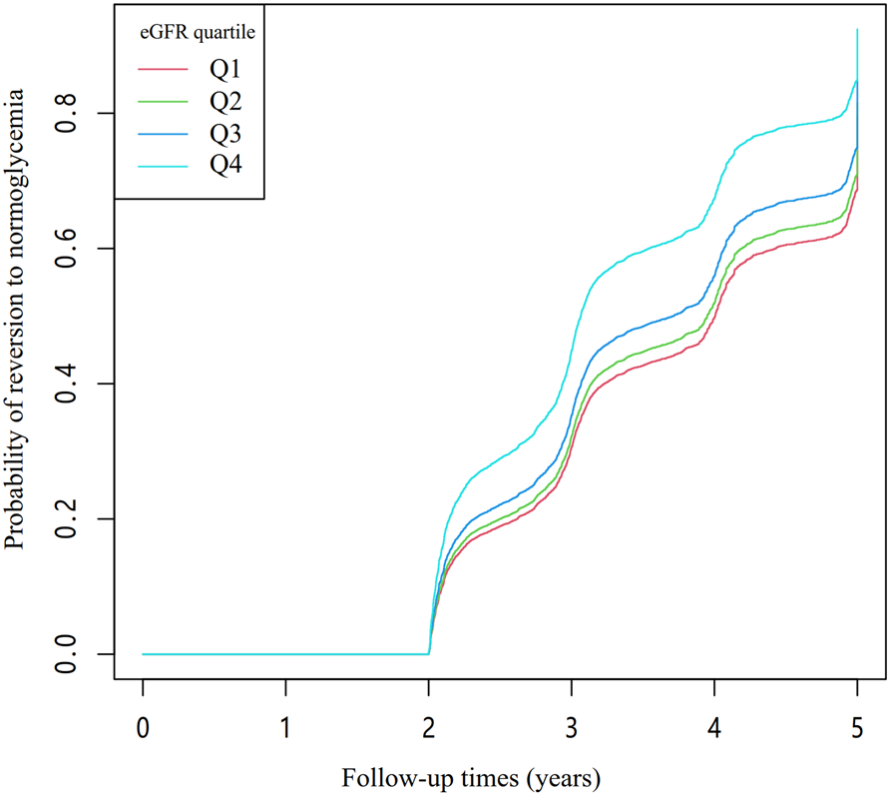
Kaplan–Meier curves for the probability of reversion to normoglycemia from IFG. This figure displays the Kaplan–Meier curves that illustrate the likelihood of reversion to normoglycemia from IFG, categorized by quartiles of eGFR. The findings reveal a gradual escalation in the probability of reversion to normoglycemia as eGFR increases, signifying that individuals with the highest eGFR exhibit the most substantial potential for transitioning from IFG to normoglycemia

Figure 4 displays Kaplan–Meier curves illustrating the likelihood of returning to normoglycemia from IFG across various eGFR categories. The log-rank test revealed a statistically significant difference in the probability of reversion to normoglycemia among the eGFR groups (*p* < 0.001). Notably, the likelihood of returning to normoglycemia increased progressively with higher eGFR values, indicating that patients with elevated eGFRs had a greater probability of reverting from IFG to normoglycemia.

### The relationship between eGFR and reversion to normoglycemia from IFG

We used multivariate Cox proportional-hazards models to explore the association between eGFR and reversion to normoglycemia in patients with IFG (Table 4). Three models were constructed to assess this relationship. In Model I (unadjusted), each incremental increase of 1 mL/min·1.73 m^2^ in eGFR was associated with a 1.3% increase in the probability of reversion to normoglycemia (HR = 1.013, 95% CI 1.011–1.014), indicating statistical significance. In Model II (minimally adjusted), which only adjusted for demographic variables, each additional ml/min·1.73 m^2^ of eGFR increased the likelihood of reversion to normoglycemia by 0.9% (HR = 1.009, 95%CI 1.008–1.011). The present study has demonstrated a statistically significant association between eGFR and the reversion to normoglycemia from IFG. In Model III, which was fully adjusted, an increase of one ml/min·1.73 m^2^ in eGFR was found to correspond to a 0.8% increase in the probability of reversion to normoglycemia (HR = 1.008, 95%CI 1.006–1.009). The reliable nature of this relationship is supported by the confidence intervals presented in Table 4.

**Table 4:** Relationship between eGFR and reversion to normoglycemia in patients with IFG in different models

Exposure	Model I (HR, 95%CI, *P*)	Model II (HR, 95%CI, *P*)	Model III (HR, 95%CI, *P*)	Model IV (HR, 95%CI, *P*)
eGFR	1.013 (1.011, 1.014) < 0.0001	1.009 (1.008, 1.011) < 0.0001	1.008 (1.006, 1.009) < 0.0001	1.007 (1.006, 1.008) < 0.0001
eGFR (SD)	1.219 (1.195, 1.243) < 0.0001	1.158 (1.135, 1.181) < 0.0001	1.126 (1.102, 1.150) < 0.0001	1.118 (1.094, 1.142) < 0.0001
eGFR Quartile				
Q1	Ref.	Ref.	Ref.	Ref.
Q2	1.063 (1.004, 1.125) 0.0353	1.025 (0.969, 1.085) 0.3897	1.011 (0.954, 1.070) 0.7190	1.015 (0.959, 1.075) 0.6066
Q3	1.188 (1.124, 1.255) < 0.0001	1.116 (1.055, 1.180) 0.0001	1.072 (1.013, 1.135) 0.0164	1.070 (1.011, 1.134) 0.0197
Q4	1.619 (1.537, 1.707) < 0.0001	1.423 (1.348, 1.502) < 0.0001	1.314 (1.241, 1.391) < 0.0001	1.295 (1.222, 1.372) < 0.0001
*P* for trend	< 0.0001	< 0.0001	< 0.0001	< 0.0001

Model I: we did not adjust other covariates

Model II: we adjust BMI, SBP, gender, family history of diabetes, DBP, smoking and drinking status

Model III: we adjust gender, SBP, BMI, FPG, family history of diabetes, DBP, BUN, TG, AST, LDL-c, ALT, HDL-c, smoking and drinking status

Model IV: we adjusted gender, family history of diabetes, BMI (smooth), TG (smooth), DBP (smooth), FPG (smooth), SBP (smooth), BUN (smooth), HDL-c (smooth), ALT (smooth), LDL-c (smooth), AST (smooth), smoking and drinking status

*HR* Hazard ratios, *CI* confidence, *Ref.* reference, *eGFR* estimated glomerular filtration rate (mL/min·1.73 m^2^), *SD* standard deviation

Since the wider range of eGFR in the present study was 55.0–151.1 ml/min·1.73 m^2^, each 1 ml/min·1.73 m^2^ change in eGFR may have a non-significant effect on the reversal of normoglycemia. To better demonstrate the effect of changes in eGFR on the reversal of normoglycemia, we added the effect of each 1 SD change in eGFR on the reversal of normoglycemia in Table 4. It was found that after adjusting for relevant confounding variables, the the probability of reversion to normoglycemia increased by 12.6% for each 1 standard deviation increase in eGFR (HR = 1.126, 95%CI 1.102–1.150).

### The results of competing risks multivariate Cox proportional-hazards regression

Table 5 displays the results of the competing analysis when progression to incident diabetes from IFG was considered. In Model I (unadjusted), we observed a positive relationship between eGFR and the probability of reversion to normoglycemia (SHR = 1.012, 95% CI 1.011–1.014). In Model II (minimally adjusted), which included adjustments for family history of diabetes, gender, SBP, BMI, DBP, smoking, and drinking status, we did not observe significant changes in the result (SHR: 1.009, 95% CI 1.008–1.010). The fully adjusted model (Model III), which also included adjustments for TG, gender, BUN, BMI, FPG, ALT, DBP, AST, family history of diabetes, HDL-c, SBP, LDL-c, smoking and drinking status, showed a positive association between eGFR and reversion to normoglycemia (SHR = 1.008, 95% CI 1.006–1.009).

**Table 5:** Relationship between eGFR and reversion to normoglycemia in patients with IFG in different models with competing risk of progression to diabetes

Exposure	Model I (SHR, 95%CI, *P*)	Model II (SHR, 95%CI, *P*)	Model III (SHR, 95%CI, *P*)
eGFR	1.012 (1.011, 1.014) < 0.0001	1.009 (1.008, 1.010) < 0.0001	1.008 (1.006, 1.009) < 0.0001
eGFR Quartile			
Q1	Ref.	Ref.	Ref.
Q2	1.063 (1.004, 1.125) 0.0353	1.024 (0.967, 1.084) 0.4138	1.010 (0.954, 1.070) 0.7295
Q3	1.188 (1.124, 1.255) < 0.0001	1.114 (1.053, 1.178) 0.0002	1.072 (1.012, 1.134) 0.0171
Q4	1.619 (1.537, 1.706) < 0.0001	1.417 (1.342, 1.496) < 0.0001	1.311 (1.239, 1.388) < 0.0001
*P* for trend	< 0.0001	< 0.0001	< 0.0001

Model I: we did not adjust other covariates

Model II: we adjust BMI, gender, SBP, family history of diabetes, DBP, smoking and drinking status

Model III: we adjust gender, SBP, BMI, DBP, BUN, FPG, TG, AST, LDL-c, ALT, family history of diabetes, HDL-c, smoking and drinking status

*SHR* subdistribution hazard ratios, *CI* confidence, *Ref*. reference

### Sensitivity analysis

To verify the robustness of our findings, we conducted a series of sensitivity analyses. First, we transformed eGFR into quartiles and incorporated it into our model as a categorical variable. The results of Tables 4 and 5 show that the HRs between groups were not entirely equivalent, indicating a possible non-linear relationship between eGFR and the probability of reversion to normoglycemia.

A GAM was employed to incorporate the continuity covariate as a curve in the equation. The outcome of Model IV in Table 4 exhibited a high degree of consistency with the fully adjusted model (HR = 1.007, 95% CI 1.006–1.008, *P* < 0.0001). *E* values were calculated to evaluate the susceptibility to unmeasured confounding, and the resultant *E* value of 1.08 suggested that the influence of unmeasured or unknown confounders on the correlation between eGFR and the probability of returning to normoglycemia was insignificant.

Furthermore, we undertook several additional sensitivity analyses. We excluded patients with eGFR < 90 mL/min·1.73 m^2^ (*N* = 19,995) and observed a positive association between eGFR and the probability of reversion to normoglycemia (HR = 1.011, 95% CI 1.010–1.013) (Table 6). We also excluded participants with a history of smoking or drinking and still observed a positive association between eGFR and reversion to normoglycemia after adjusting for confounding factors (Table 6).

**Table 6:** Relationship between eGFR and the probability of reverting from IFG to normoglycemia in different sensitivity analyses

Exposure	Model a (HR, 95% CI, *P*)	Model b (HR, 95% CI, *P*)	Model c (HR, 95% CI, *P*)	Model d (HR, 95% CI, *P*)
eGFR	1.011 (1.010, 1.013) < 0.0001	1.008 (1.006, 1.009) < 0.0001	1.009 (1.007, 1.010) < 0.0001	1.008 (1.006, 1.009) < 0.0001
eGFR (Quartile)				
Q1	Ref.	Ref.	Ref.	Ref.
Q2	1.029 (0.941, 1.126) 0.5265	1.018 (0.951, 1.090) 0.6020	1.031 (0.967, 1.099) 0.3452	1.010 (0.954, 1.070) 0.7234
Q3	1.094 (1.000, 1.196) 0.0499	1.102 (1.030, 1.178) 0.0045	1.112 (1.043, 1.185) 0.0011	1.073 (1.014, 1.136) 0.0148
Q4	1.338 (1.223, 1.463) < 0.0001	1.301 (1.217, 1.390) < 0.0001	1.372 (1.288, 1.462) < 0.0001	1.317 (1.244, 1.394) < 0.0001
*P* for trend	< 0.0001	< 0.0001	< 0.0001	< 0.0001

Model a was a sensitivity analysis conducted on 19,995 participants without eGFR < 90 mL/min·1.73 m^2^. We adjusted for SBP, family history of diabetes, BMI, gender, DBP, FPG, TG, ALT, HDL-c, BUN, AST, LDL-c, smoking and drinking status

Model b was a sensitivity analysis performed on 17,848 participants who had never smoked. We adjusted for SBP, family history of diabetes, BMI, gender, DBP, FPG, TG, ALT, HDL-c, BUN, AST, LDL-c, and drinking status

Model c was a sensitivity analysis conducted on 19,952 participants who had never consumed alcohol. We adjusted for SBP, family history of diabetes, BMI, gender, DBP, FPG, TG, ALT, HDL-c, BUN, AST, LDL-c, and smoking status

Model d was a sensitivity analysis carried out on 173,301 participants without adjusting for smoking and drinking status. We adjusted for SBP, family history of diabetes, BMI, gender, DBP, FPG, TG, ALT, HDL-c, BUN, AST, LDL-c

*HR* hazard ratios, *Ref*. reference, *CI* confidence, *eGFR* estimated glomerular filtration rate (mL/min·1.73 m^2^)

However, due to the high percentage of missing data (around 70%) for smoking and alcohol status, we excluded these variables as covariates in some sensitivity analyses. Even so, the results remained similar to those of previous analyses (HR = 1.008, 95% CI 1.006–1.010).

### Nonlinear relationship between eGFR and reversion to normoglycemia

**Fig. 5:**
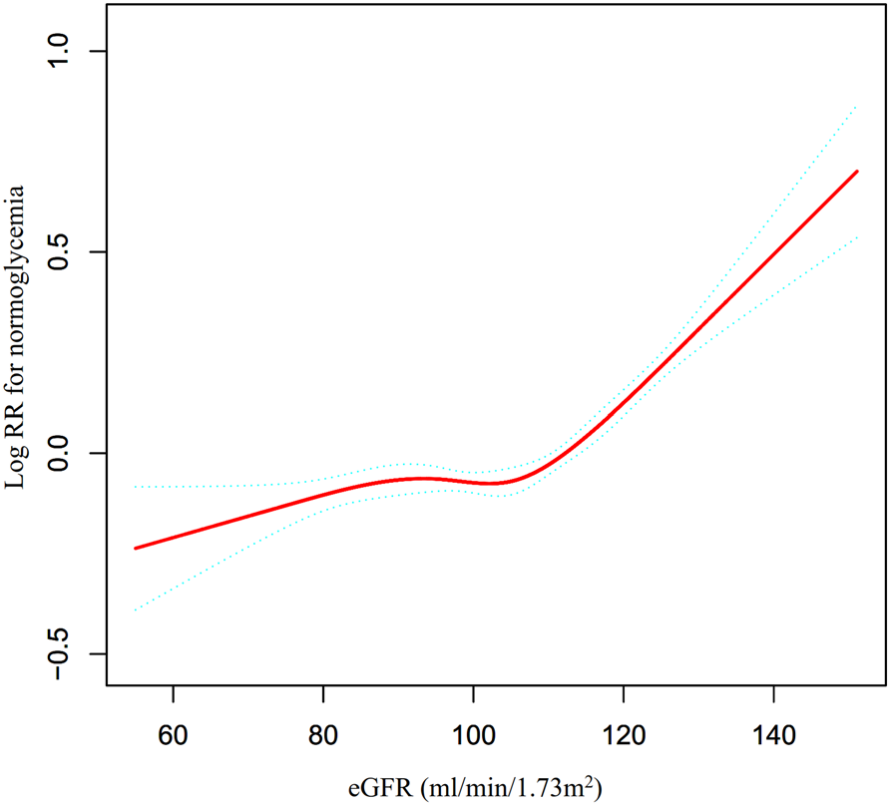
Nonlinear relationship between eGFR and reversion to normoglycemia in patients with IFG. This figure features a Cox proportional hazards model with cubic spline functions that we utilized to examine the relationship between eGFR and the probability of reversion from IFG to normoglycemia. The results indicate a nonlinear correlation between eGFR and this probability, with an inflection point of 111.962 ml/min·1.73 m^2^

A Cox proportional hazards model with cubic spline functions was employed to examine the correlation between eGFR and the likelihood of reversal to normoglycemia in individuals with IFG. The findings indicated a non-linear connection, as depicted in Fig. 5. To delve deeper into this association, a standard binary two-piecewise Cox proportional hazards model was applied to the data, and the optimal fit was determined through the log-likelihood ratio test. The test yielded a *P* value of less than 0.05, as presented in Table 7.

**Table 7:** Result of the two-piecewise Cox regression model

reversion to normoglycemia	Model* (HR, 95%CI, *P*)	Model^**#**^ (HR, 95%CI, *P*)
Fitting model by standard Cox regression	1.008 (1.006, 1.009) < 0.0001	1.011 (1.010, 1.013) < 0.0001
Fitting model by two-piecewise Cox regression		
Inflection point of eGFR	111.962	112.029
≤ Inflection point	1.003 (1.001, 1.005) 0.0064	1.005 (1.001, 1.008) 0.0161
> Inflection point	1.019 (1.015, 1.022) < 0.0001	1.018 (1.015, 1.022) < 0.0001
*P* for log-likelihood ratio test	< 0.001	< 0.001

Model *: analysis among all participants; Model I#: sensitivity analysis in participants without eGFR < 90 mL/min·1.73 m^2^ (N = 19,995)

We adjusted SBP, family history of diabetes, BMI, gender, DBP, FPG, TG, ALT, HDL-c, BUN, AST, LDL-c, smoking and drinking status

HR, Hazard ratios; Ref reference; CI confidence, eGFR, estimated glomerular filtration rate (mL/min·1.73 m^2^)

By employing a recursive algorithm, we determined the inflection point to be 111.962 ml/min·1.73 m^2^ through the utilization of two-piecewise Cox proportional-hazards models. Subsequently, we obtained HR and CI values on either side of this inflection point. On the left-hand side, the HR and 95%CI were 1.003 (1.001, 1.005), respectively, while on the right-hand side, they were 1.019 (1.015, 1.022), respectively.

**Fig. 6:**
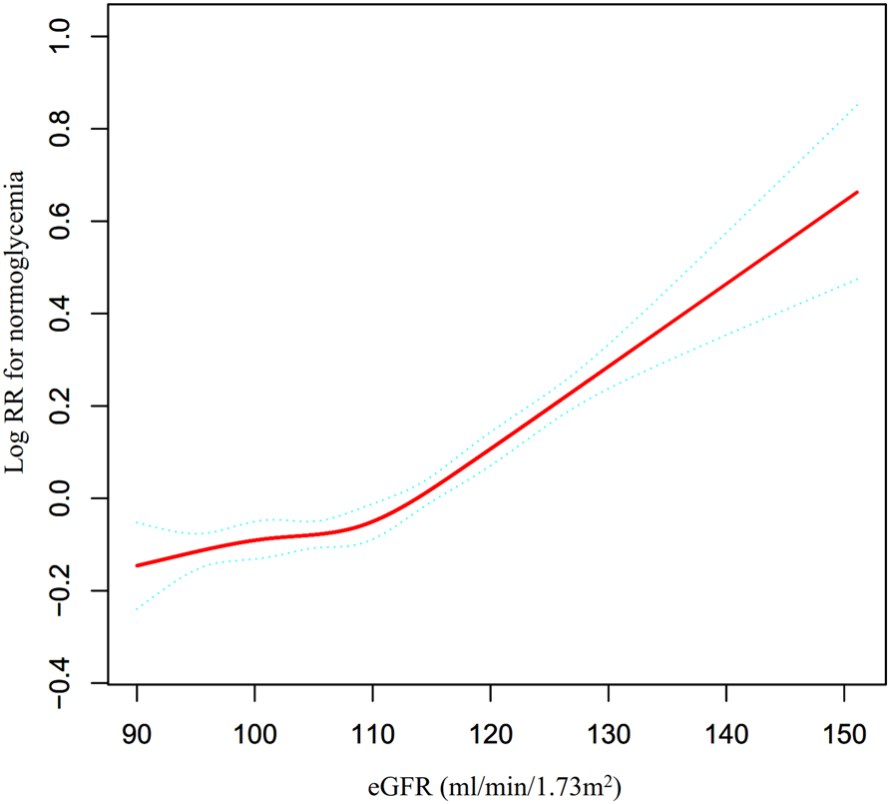
Nonlinear relationship between eGFR and reversion to normoglycemia from IFG in participants with eGFR ≥ 90 mL/min·1.73 m^2^. This figure depicts the utilization of a Cox proportional hazards model incorporating cubic spline functions to evaluate the correlation between eGFR and the likelihood of returning to normoglycemia from IFG in subjects possessing an eGFR of ≥ 90 mL/min·1.73 m^2^. The results demonstrate that, even within this subset, the association between eGFR and reversion to normoglycemia from IFG remained non-linear, with an inflection point of 112.029 ml/min·1.73 m^2^

To test the robustness of our findings, we conducted a sensitivity analysis and excluded patients with eGFR < 90 mL/min·1.73 m^2^. Despite this exclusion, a non-linear correlation between eGFR and the likelihood of returning to normoglycemia (as depicted in Fig. 6) was still observed, with an inflection point of 112.029 ml/min·1.73 m^2^. The HR and 95%CI were 1.005 (1.001, 1.008) and 1.018 (1.015, 1.022) on the left and right sides of the inflection point, respectively (as shown in Table 7).

### Results of subgroup analysis

We evaluated the interaction between various variables and the association between eGFR and the probability of reversal to normoglycemia in both prespecified and exploratory subgroups (Table 8). Our analyses revealed no significant interactions for age, SBP, BMI, DBP, TG, gender, or family history of diabetes. However, we did observe significant interactions with one variable: drinking status.

**Table 8:** Stratified associations between eGFR and reversion to normoglycemia in patients with IFG in prespecified and exploratory subgroups

Characteristic	No of participants	HR (95%CI)	*P* value	*P* for interaction
Age (years)				0.4975
< 60	18,383	1.005 (1.003, 1.007)	< 0.0001	
≥ 60	6158	1.001 (0.999, 1.003)	0.0580	
Gender				0.2182
Male	16,265	1.007 (1.005, 1.009)	< 0.0001	
Female	8276	1.009 (1.006, 1.011)	< 0.0001	
BMI (kg/m^2^)				0.5534
< 18.5	470	1.006 (0.999, 1.013)	0.1023	
≥ 18.5, < 24	9600	1.008 (1.006, 1.010)	< 0.0001	
≥ 24, < 28	10,417	1.007 (1.005, 1.009)	< 0.0001	
≥ 28	4054	1.005 (1.002, 1.009)	0.0043	
Smoking status				0.6506
Never smoker	17,848	1.008 (1.006, 1.009)	< 0.0001	
Ever smoker	1074	1.008 (1.001, 1.015)	0.0191	
Current smoker	5619	1.006 (1.003, 1.009)	< 0.0001	
Drinking status				0.0005
Never drinker	19,952	1.009 (1.007, 1.010)	< 0.0001	
Ever drinker	3749	1.001 (0.998, 1.005)	0.4632	
Current drinker	840	1.005 (0.997, 1.013)	0.2524	
Family history of diabetes				0.2974
No	23,923	1.008 (1.006, 1.009)	< 0.0001	
Yes	618	1.002 (0.993, 1.012)	0.6061	
SBP (mmHg)				0.2909
< 140	19,347	1.008 (1.006, 1.009)	< 0.0001	
≥ 140	5194	1.006 (1.003, 1.009)	< 0.0001	
DBP (mmHg)				0.2331
< 90	20,999	1.008 (1.006, 1.009)	< 0.0001	
≥ 90	3542	1.005 (1.001, 1.009)	0.0066	
TG (mmol/L)				0.9203
< 1.7	15,011	1.008 (1.006, 1.009)	< 0.0001	
≥ 1.7	9530	1.007 (1.005, 1.010)	< 0.0001	

Note 1: Above model adjusted for SBP, family history of diabetes, BMI, gender, DBP, FPG, TG, ALT, HDL-c, BUN, AST, LDL-c, drinking status, and smoking status

Note 2: In each case, the model is not adjusted for the stratification variable

*HR* hazard ratios, *Ref*. reference, *CI* confidence

Specifically, a stronger association between eGFR and the probability of reversal to normoglycemia was observed in the participants who had never drunk (HR = 1.009, 95% CI 1.007–1.010). In contrast, the association of eGFR with the probability of reversal to normoglycemia from IFG was attenuated among participants who ever (HR = 1.001, 95% CI 0.998–1.005) or currently drunk (HR = 1.005, 95% CI 0.997–1.013).

## Discussion

The objective of this retrospective cohort study was to examine the correlation between eGFR and the reversal of normoglycemia in individuals with IFG. Our analysis demonstrated a noteworthy correlation between an elevation in eGFR and an increased likelihood of regression to normoglycemia. In addition, we observed a threshold effect curve, which indicated varying associations between eGFR and reversion to normoglycemia on either side of the inflection point. Moreover, we identified drinking status as a potential moderator of this relationship, with never-drinkers exhibiting significantly stronger associations.

In a prospective cohort study of 491 participants, 22.6% with pre-diabetes returned to normoglycemia over a median follow-up of 2.5 years [45]. Another study reported that 54% of participants had returned to normoglycemia after 1 year, while 6% had developed diabetes [15]. Similarly, a Chinese cohort study of 14,231 adults found that 44.9% of those with pre-diabetes reverted to normoglycemia within 2 years [46]. In our study, we followed patients with IFG for 5 years and observed a return to normoglycemia in 45.70% of cases. These variations in reversion rates between studies may be due to differences in participant age, duration of follow-up, and ethnic background. Nonetheless, all studies confirm that a significant fraction of individuals with IFG can revert to normoglycemia. Therefore, identifying contributing factors to this reversion is crucial for preventing diabetes and its complications.

Prior research has investigated the association between eGFR and the risk of diabetes; however, the majority of these investigations were not carried out among the Chinese populace [47–50]. A prospective cohort study comprising 1713 American participants with decreased renal function and no diabetes at baseline revealed a significant association between CKD and a higher incidence of T2DM compared to the general population [51]. Likewise, a population-based cohort study conducted in Taiwan identified CKD as a crucial and independent predictor of diabetes, with an adjusted HR of 1.204 and a 95% CI of 1.11 to 1.31[52]. Our previous research also discovered that decreased eGFR was strongly linked to a higher risk of developing both diabetes (HR = 0.986, 95% CI 0.984, 0.988) [20] and pre-diabetes (HR = 0.993, 95% CI 0.992–0.995) [21] in the general Chinese population. Given this information, we posited that a diminished eGFR might be linked to a decreased likelihood of returning to normoglycemic levels from a state of IFG. However, no studies have previously explored this relationship. Our new study has confirmed that elevated eGFR is indeed linked with a higher probability of reversal to normoglycemia in patients with IFG. We also found that this positive association remained stable when we excluded smoking and drinking status from the multiple regression equation. Sensitivity analysis showed that the relationship was consistent for participants who neither smoked nor drank and those without an eGFR < 90 mL/min/1.73 m^2^. These findings have important clinical implications: clinicians should consider interventions to maintain eGFR levels as a means of promoting regression to normoglycemia from IFG. Clinically, the positive correlation between eGFR and the reversal of normoglycemia suggests that in patients with IFG, the possibility of reversal of normoglycemia may be increased by slowing down the progression of renal function, effectively preventing further deterioration of glycemia.

To our knowledge, our study is the initial observation of a non-linear correlation between eGFR and reversion to normoglycemia in patients with IFG. Our analysis employed a two-piecewise Cox proportional hazards model to elucidate this non-linear relationship, with an inflection point of 111.962 ml/min/1.73 m^2^ after controlling for confounding variables. The results of our study demonstrate that a decrease in eGFR below 111.962 ml/min/1.73m^2^ is associated with a reduced likelihood of reversal to normoglycemia, with a decline of 0.3% for every 1 ml/min/1.73 m^2^ decrease in eGFR (HR = 1.003, 95% CI 1.001–1.005). Conversely, when eGFR is above 111.962 ml/min/1.73 m^2^, a decrease of 1 unit in eGFR is associated with a 1.9% decrease in the probability of reversing to normoglycemia (HR = 1.019, 95% CI 1.015–1.022). Therefore, a reduction in eGFR levels in individuals with IFG is associated with a reduction in the likelihood of reversion to normoglycemia. However, the rate of decline in the likelihood of reversal to normoglycemia was faster when eGFR exceeded 111.962 ml/min/1.73 m^2^ and slower when eGFR was below 111.962 ml/min/1.73 m^2^. Insulin resistance (IR) has been shown to play a crucial role in the regression and progression of pre-diabetes [27], and a recent study confirmed that a decrease in eGFR is associated with an increase in IR [47]. Moreover, additional research has indicated that the correlation between eGFR and the risk of diabetes is non-linear in instances, where eGFR falls below 80 ml/min/1.73 m^2^. Specifically, the likelihood of developing diabetes increases and subsequently decreases as eGFR rises [48]. Several studies have suggested that a decreased eGFR is associated with a higher cumulative probability of developing diabetes, particularly when eGFR levels are less than 60 ml/min/1.73 m^2^ either at baseline or as time progresses [51]. A separate study demonstrated that for each 10 ml/min/1.73 m^2^ decrease in eGFR from an eGFR level greater than 90 ml/min/1.73 m^2^, there was a 2.2% increase in fasting insulin concentration (95% CI, 1.4–2.9%; *P* < 0.001) and a 1.1% reduction in insulin sensitivity index (95% CI, 0.03–2.2%; *P* = 0.04) [47]. Interestingly, a decrease in eGFR has also been associated with an increased B-cell function index, lower 2-h glucose levels, and a decreased risk of glucose intolerance [47]. In our prior research, it was observed that there was no statistically significant correlation between incident diabetes and eGFR within the range of 60–98.034 ml/min per 1.73 m^2^ [20]. We hypothesized that the observed phenomenon could be attributed to the decline in eGFR, resulting in an elevated risk of diabetes and decreased insulin sensitivity. In addition, the reduced eGFR was found to be associated with increased insulin levels and enhanced B-cell function within the body. These findings provide a potential explanation for the relatively modest correlation between eGFR and the restoration of normoglycemia, particularly when eGFR falls below 111.962 ml/min/1.73 m^2^.

In a study conducted by Lorenzo et al., a U-shaped relationship between eGFR and the risk of T2DM was identified. The researchers discovered that individuals falling within the upper and lower ranges of GFR had a heightened risk of developing diabetes in the future [48]. The participants in their study exhibited eGFR levels ranging from 39.9 to 239.1 ml/min/1.73 m^2^. Notably, they observed an increased risk of T2DM among participants with eGFR levels below 65 ml/min/1.73 m^2^ and above 100 ml/min/1.73 m^2^ [48]. The eGFR range in our study ranged from 54.98 to 151.09 ml/min/1.73 m^2^. We observed a stronger positive correlation between eGFR and the restoration of normoglycemia in participants with eGFR levels above 111 ml/min/1.73 m^2^. This enhanced positive association can be attributed to two key factors. First, as renal function is at a higher level during this period, the decline in eGFR is predominantly characterized by insulin resistance rather than impaired insulin excretion. Consequently, the reduction in eGFR has a more substantial impact on decreasing the likelihood of restoring normoglycemia. Second, the involvement of glomerular hyperperfusion further contributes to the decline in renal function [53].

Drawing from the literature reports cited, it is posited that a correlation exists between reduced eGFR and heightened susceptibility to diabetes, reduced insulin sensitivity, elevated insulin levels, and amplified B-cell function. The interplay between eGFR and glucose sensitivity is intricate, given that individuals with impaired eGFR exhibit underlying pathologies and intrinsic distinctions from those with unimpaired renal function.

The identification of a curvilinear association between eGFR and the reversion to normoglycemia in individuals with IFG holds significant clinical significance. It can guide clinical consultations and aid in decision-making for optimizing diabetes prevention strategies. Prior studies have demonstrated that a short-term restoration of normoglycemia effectively mitigates the likelihood of developing T2DM among individuals with prediabetic conditions [12]. Hence, the management of IFG ought to strive towards achieving normoglycemia as opposed to solely mitigating its potential consequences and reducing the probability of progression to type 2 diabetes mellitus.

Hence, it may be beneficial for patients with IFG and different renal function statuses to delay the progression of renal decompensation to maintain a higher likelihood of achieving normoglycemia reversal. This is particularly crucial for patients with an eGFR above 111 ml/min/1.73 m^2^ in the early stages of renal injury. It is important to remain vigilant regarding the potential decline in eGFR due to factors, such as proteinuria or glomerular hyperfiltration. Such declines can significantly impact the likelihood of achieving normoglycemia reversal and ultimately increase the risk of developing diabetes mellitus. These findings provide valuable insights into improving the chances of returning to normoglycemia from IFG for individuals with varying levels of renal function. Furthermore, preserving renal function may represent a novel therapeutic approach to reduce the risk of diabetes.

Upon conducting a subgroup analysis, it was discovered that the association between eGFR and the probability of reversion to normoglycemia might be influenced by drinking status, with a more pronounced correlation observed in individuals who have abstained from alcohol consumption. Previous studies have indicated that drinking is linked to insulin resistance and heightened susceptibility to diabetes [41, 54]. In addition, our study found that drinking was associated with a decreased likelihood of reversion to normoglycemia. Therefore, it is not surprising that the association between eGFR and the likelihood of reversion to normoglycemia is weakened in individuals who currently or have ever consumed alcohol due to the influence of drinking. Given that drinking status may modify the relationship between eGFR and the likelihood of reversion to normoglycemia, it is clinically feasible to increase the likelihood of reversion by controlling or reducing alcohol consumption in at-risk individuals. This highlights the importance of lifestyle modifications, such as reducing drinking to optimize pre-diabetes management and improve patient outcomes.

This study has several noteworthy strengths. First, it is the first study to explore the relationship between eGFR and reversion to normoglycemia from IFG, specifically in the Chinese population. Second, this study's identification of inflection points and the non-linear association between eGFR and recovery from IFG to normoglycemia constitutes a significant contribution to the field. Third, a multiple imputation approach was utilized to mitigate the impact of missing covariate information, thereby maximizing statistical power and minimizing bias. Fourth, a set of sensitivity analyses were executed to ascertain the dependability of the results. Furthermore, a multivariate Cox proportional hazards model of competing risks was utilized, with the development of IFG to diabetes as the competing risk for reversing normoglycemia.

The study has several limitations that are worth considering. First, given the homogeneity of the study population being exclusively Chinese, additional inquiry is warranted to ascertain the correlation between eGFR and the restoration of normoglycemia in those with IFG who possess distinct genetic backgrounds. Second, while IFG serves as a marker for pre-diabetes, it inadequately captures the intricacies of the ailment. Since in early stage diabetes, initial stages of hyperglycemia are usually postprandial and are thus best captured via 2 h OGTT, this greatly limits the study’s definition of “normoglycemia”. In addition, the diagnosis of prediabetes and diabetes cannot be ascertained with just 1 FPG measurement as these lab tests can be spurious and can be affected by many factors. There should be at least 2 FPG measurements taken at least 2 weeks apart. Although the multiple measurements of FPG and the measurement of 2-h oral glucose tolerance tests or HbA1C levels present challenges in the context of a sizable study cohort, our objective is to tackle this issue in forthcoming investigations or collaborate with external entities to obtain this data. Third, early stage CKD in diabetes is usually characterized first by proteinuria before any noticeable reduction in eGFR. We should look at proteinuria in addition to eGFR to better capture these early insults to the kidney. In the future, we can consider designing our studies or collaborating with other researchers to collect as many variables as possible, including information on proteinuria. Fourth, due to the secondary nature of the analysis, it was not feasible to account for variables that were not initially incorporated in the data set, such as insulin concentration, waist circumference, physical activity/exercise, diet, medications, and comorbidities, such as pre-existing kidney disease and urinary tract infection. Nevertheless, the *E* value computation was employed to ascertain that unobserved confounding factors were improbable to elucidate the findings. Fifth, this observational study conducted post hoc has established a correlation between eGFR and the regression of normoglycemia in patients with IFG, indicating an association rather than a causal relationship. In addition, the study evaluated eGFR and other parameters at baseline without considering their variations over time. Future studies will be structured to gather multiple data points, including physical activity/exercise, diet, medications, comorbidities, such as pre-existing kidney disease and urinary tract infection, and details on the changes in eGFR during patient follow-up or through collaboration with other researchers.

## Conclusion

The present study provides evidence of an autonomous correlation between eGFR and the regression to normoglycemia in Chinese adults with IFG. Furthermore, a non-linear association and threshold effect were detected between eGFR and normoglycemia. These results furnish significant insights into enhancing the probability of returning to normoglycemia from IFG in individuals with varying renal function statuses in the future. In addition, preserving renal function may represent a novel therapeutic approach to attenuate the risk of diabetes.

### Supplementary Information

**Additional file 1: Table S1.** Collinearity diagnostics steps.

## Data Availability

The 'DATADRYAD' database (https://datadryad.org/stash) offers access to the data.

## Abbreviations

IDF: International Diabetes Federation
CKD–EPI: Chronic Kidney Disease–Epidemiology Collaboration
T2DM: Type 2 diabetes mellitus
SBP: Systolic blood pressure
HbA1c: Glycated hemoglobin A1c
ALT: Alanine aminotransferase
TG: Triglyceride
BMI: Body mass index
Scr: Serum creatinine
DBP: Diastolic blood pressure
DM: Diabetes mellitus
eGFR: Estimated glomerular filtration rate
GAM: Generalized additive models
LDL-c: Low-density lipid cholesterol
BUN: Blood urea nitrogen
VIF: Variance inflation factor
CKD: Chronic kidney disease
HDL-c: High-density lipoprotein cholesterol
AST: Aspartate aminotransferase
FPG: Fasting plasma glucose
HR: Hazard ratios
IGT: Impaired glucose tolerance
TC: Total cholesterol
Ref: Reference
IR: Insulin resistance
MAR: Missing-at-random
CI: Confidence intervals
IFG: Impaired fasting glucose
SD: Standard deviation
