# Supplementary material for: Association between estimated glomerular filtration rate and reversion to normoglycemia in people with impaired fasting glucose: a 5-year retrospective cohort study
Source: Eur J Med Res. 2024 Feb 22;29:140. doi: 10.1186/s40001-024-01669-y (PMC10882936; doi:10.1186/s40001-024-01669-y)
Supplement: Supplementary file 1 — Additional file 1: Table S1. Collinearity diagnostics steps. [file 40001_2024_1669_MOESM1_ESM.docx]

Association between estimated glomerular filtration rate and reversion to normoglycemia in people with impaired fasting glucose: a 5-year retrospective cohort study

**Running title:** **eGFR and reversion to normoglycemia**

**Lirong Tu^1#^,** **Haofei Hu^2,3#^****,** **Xinglei Zhou^4^, Heping Zhang^1^, Xiaohui Liu^1*^, Dehua Yang^5*^, Yongcheng He^6,1*^**

^1^Department of Nephrology, Affiliated Hospital of North Sichuan Medical College, Nanchong 637000, Sichuan Province, China

^2^Department of Nephrology, The First Affiliated Hospital of Shenzhen University, Shenzhen 518000, Guangdong Province, China

^3^Department of Nephrology, Shenzhen Second People’s Hospital, Shenzhen 518000, Guangdong Province, China

^4^Department of Nephrology, Second Affiliated Hospital of Xuzhou Medical University, Xuzhou 221006, Jiangsu Province, China

^5^Department of Pediatrics, Shenzhen Hengsheng Hospital, Shenzhen 518103, Guangdong Province, China

^6^Department of Nephrology, Shenzhen Hengsheng Hospital, Shenzhen 518103, Guangdong Province, China

**^#^ Lirong Tu and Haofei Hu have contributed equally to this work.**

***Corresponding authors**

**Xiaohui Liu**

Department of Nephrology,

Affiliated hospital of North Sichuan Medical College,

No.1 Maoyuan South Rd,

Nanchong 637000,

Sichuan Province,

China

E-mail: [15328899878@163.com](mailto:15328899878@163.com)

***Corresponding authors**

**Dehua Yang**

Department of Pediatrics

Shenzhen Hengsheng Hospital,

No. 20 Yintian Road, Baoan District,

Shenzhen 518103,

Guangdong Province,

China

E-mail: [yangdehua2023@126.com](mailto:yangdehua2023@126.com)

***Corresponding author**

Yongcheng He

Department of Nephrology,

Shenzhen Hengsheng Hospital,

No. 20 Yintian Road, Baoan District,

Shenzhen 518103,

Guangdong Province,

China

E-mail: heyongcheng640815@126.com

**Table S1. Collinearity diagnostics steps.**

| Variable | VIF  Step 1 | Step 2 |
| --- | --- | --- |
|  |  |  |
| Gender | 1.4 | 1.4 |
| Smoking status | 1.3 | 1.3 |
| Drinking status | 1.2 | 1.2 |
| ALT(U/L) | 3.2 | 3.2 |
| AST(U/L) | 3.0 | 3.0 |
| Family history | 1.0 | 1.0 |
| BUN (mmol/L) | 1.1 | 1.1 |
| FPG (mmol/L) | 1.1 | 1.1 |
| TC (mmol/L) | 6.4 | NA |
| TG (mmol/L) | 1.8 | 1.2 |
| HDL-c(mmol/L) | 1.4 | 1.2 |
| LDL-c(mmol/L) | 5.3 | 1.1 |
| BMI (kg/m^2^) | 1.3 | 1.3 |
| SBP (mmHg) | 1.8 | 1.8 |
| DBP (mmHg) | 1.8 | 1.8 |

BMI, Body mass index; SBP, Systolic blood pressure; DBP, Diastolic blood pressure; ALT, Alanine aminotransferase; AST, Aspartate aminotransferase; TC, Total cholesterol; TG, Triglyceride; HDL-c, High-density lipoprotein cholesterol; LDL-c, Low-density lipid cholesterol; BUN, Serum urea nitrogen; FPG, Fasting plasma glucose;

Abbreviation: VIF: variance inflation factor; VIF = 1/(1-R^2^).

Note: The variables with VIF>5 will be regarded as collinear variables and cannot be included in the multiple regression model.
